# Deep Learning of Simultaneous Intracranial and Scalp EEG for Prediction, Detection, and Lateralization of Mesial Temporal Lobe Seizures

**DOI:** 10.3389/fneur.2021.705119

**Published:** 2021-11-11

**Authors:** Zan Li, Madeline Fields, Fedor Panov, Saadi Ghatan, Bülent Yener, Lara Marcuse

**Affiliations:** ^1^Department of Electrical, Computer, and Systems Engineering (ECSE), Rensselaer Polytechnic Institute, Troy, NY, United States; ^2^Department of Neurology, Icahn School of Medicine at Mount Sinai, New York, NY, United States; ^3^Department of Computer Science (CS) and Electrical, Computer, and Systems Engineering (ECSE), Rensselaer Polytechnic Institute, Troy, NY, United States

**Keywords:** intracranial and scalp EEG, deep neural networks, LSTM (long short term memory networks), seizure lateralization, seizure prediction, convolutional neural networks, seizure detection

## Abstract

In people with drug resistant epilepsy (DRE), seizures are unpredictable, often occurring with little or no warning. The unpredictability causes anxiety and much of the morbidity and mortality of seizures. In this work, 102 seizures of mesial temporal lobe onset were analyzed from 19 patients with DRE who had simultaneous intracranial EEG (iEEG) and scalp EEG as part of their surgical evaluation. The first aim of this paper was to develop machine learning models for seizure prediction and detection (i) using iEEG only, (ii) scalp EEG only and (iii) jointly analyzing both iEEG and scalp EEG. The second goal was to test if machine learning could detect a seizure on scalp EEG when that seizure was not detectable by the human eye (surface negative) but was seen in iEEG. The final question was to determine if the deep learning algorithm could correctly lateralize the seizure onset. The seizure detection and prediction problems were addressed jointly by training Deep Neural Networks (DNN) on 4 classes: non-seizure, pre-seizure, left mesial temporal onset seizure and right mesial temporal onset seizure. To address these aims, the classification accuracy was tested using two deep neural networks (DNN) against 3 different types of similarity graphs which used different time series of EEG data. The convolutional neural network (CNN) with the Waxman similarity graph yielded the highest accuracy across all EEG data (iEEG, scalp EEG and combined). Specifically, 1 second epochs of EEG were correctly assigned to their seizure, pre-seizure, or non-seizure category over 98% of the time. Importantly, the pre-seizure state was classified correctly in the vast majority of epochs (>97%). Detection from scalp EEG data alone of surface negative seizures and the seizures with the delayed scalp onset (the surface negative portion) was over 97%. In addition, the model accurately lateralized all of the seizures from scalp data, including the surface negative seizures. This work suggests that highly accurate seizure prediction and detection is feasible using either intracranial or scalp EEG data. Furthermore, surface negative seizures can be accurately predicted, detected and lateralized with machine learning even when they are not visible to the human eye.

## Introduction

Epilepsy is characterized by recurrent and unpredictable seizures. This unpredictability is the core of suffering in the person with epilepsy. Certain actions, like taking medication and getting enough rest, decrease the risk of seizures. However, there is never a guarantee for a seizure free day. The field, and this collection of articles, is working to address this suffering by improving the accuracy of seizure prediction, detection and forecasting. Forecasting differs from prediction by identifying a period of time, lasting hours to days, during which the person is more likely to have seizures based upon their known prior patterns and rhythms (1). The focus of our work is not forecasting but in seizure prediction and detection. In seizure prediction, the goal is to provide a warning that a seizure is about to occur within minutes. For this warning to be useful, it must be accurate with a low false positive and a low false negative rate.

The field of seizure prediction was established in the 1980s, but after >20 years, a comprehensive review published in 2007 concluded that “the current literature allows no definite conclusion as to whether seizures are predictable by prospective algorithms” (2). Nevertheless, in the past decade, several innovations have driven the field forward including the compiling of extensive databases of long-term EEG recordings; the establishment of international seizure prediction competitions; and a prospective trial of a seizure forecasting device that provided convincing evidence that forecasting of seizures is possible (3).

Several reasons can be listed for this problem to evade success including: inadequate amount of data; complexity of data generated by EEG signals (noisy, non-linear, and non-stationary); and lack of labeled data for certain classes. This is partially due to the fact that EEG signal intensity is very small, in μV range, and there are significant sensing difficulties given physiological and non-physiological artifacts.

The nature of data collected by intracranial EEG (iEEG) and scalp EEG differs greatly. Scalp EEG is readily available and is not invasive. However, it is more prone to artifacts introduced by shifting electrodes, muscle interference, and the effects of volume conduction. Intracranial EEG has a better signal-to-noise ratio than scalp EEG and can target specific areas of the brain directly. Most previous work focuses on either scalp or iEEG recordings since data sets that contain simultaneous recordings of scalp and iEEG on the same patient are exceedingly rare. The novelty of the work in this paper rests on a simultaneous iEEG and scalp EEG data sets.

There are recent comprehensive survey papers on both seizure detection and prediction (4–8) While many of the studies for seizure detection are focused on training supervised learning algorithms on EEG signals (4–6, 9), there are also unsupervised algorithms based on multiway tensor analysis of scalp EEG signals (10) and tunable Q factor wavelet transformation (11) or non-negative matrix factorization of iEEG signals (12).

Seizure detection and prediction systems using intracranial or scalp EEG signals rely on moving window analysis on extracted features to generate predictions. One of the main challenges for accurate prediction is extracting and evaluating linear and non-linear univariate and bivariate features from the signal. However, to achieve high sensitivity and a low false prediction rate, many of the previous studies relied on handcraft feature extraction and/or tailored feature extraction, which is performed for each patient independently. This approach, however, is not generalizable, and requires significant modifications (13).

The length of the pre-ictal period during which it is possible to predict the seizure is called the prediction horizon or pre-seizure period. The electrical changes that occur in the brain prior to a seizure are poorly understood and undetectable by the human eye. In the literature, the length of the pre-seizure period has varied from minutes to hours (14), and has often been left as a design choice. Estimates as to the length of the pre-seizure period exist, but the estimates are not shown to be general (14, 15) or they are patient specific (16, 17).

In our prior work, the length of the pre-seizure period was determined as a part of the learning process and optimized using grid search (18) on scalp EEG data. The pre-seizure length was validated by analyzing the extracted features with different pre-ictal lengths to elucidate the phase transition between the interictal and pre-seizure state (18). The length of the horizon was determined from the EEG data to be optimal at 10 min.

From a machine learning perspective, we built on current models and added new methodology. Previously the maximal absolute cross correlation value was defined as a functional connectivity measure and further calculated for each pair of EEG channels to quantify the similarity between any two EEG signals (19). In this work, we used 3 methods to build similarity graphs [Correlation Coefficient, Mutual Information and Waxman model (20)] and used these as input into the deep neural networks (DNN). A similarity graph is denoted by *G* = *(V, E)* where the vertex set *V* is the set of electrodes and the edge set *E* contains an edge *(i, j)* between the vertices *i* and *j* if they are “similar.” Transforming raw EEG signal to a graph representation enables us to capture spatial information as well as frequency and time domain information.

In a seizure detection model (21) using DNNs, raw EEG signal was segmented into 5 second (s) epochs to discriminate the EEG seizure from the background. We expanded on that concept and examined several window sizes ranging from 1 to 6 s and evaluated the impact of this parameter on the accuracy of the results.

For this paper, 102 seizures of mesial temporal lobe onset were analyzed from 19 patients who had simultaneous stereo and scalp EEG as part of their evaluation for drug resistant epilepsy (DRE). The first aim of this paper was to develop machine learning models for seizure prediction and detection (i) using iEEG only, (ii) scalp EEG only and (iii) jointly analyzing both iEEG and scalp EEG. The data sets allowed for a direct comparison of classification accuracy. The second goal was to test if machine learning can detect a seizure on scalp EEG when that seizure was not detectable by the human eye (surface negative) but was seen in iEEG. The final question was to determine if the deep learning algorithm could correctly lateralize the seizure onset. We tested various combinations of machine learning algorithms to determine the highest accuracy of classifying the EEG data into either non-seizure, pre-seizure or seizure (right vs. left).

## Materials and Methods

### Data Sets

The study was approved by the Icahn School of Medicine Institutional Review Board. Simultaneous iEEG and scalp EEG were collected from patients using a Natus XLTEK 128 or Natus Quantum amplifier (Natus Medical Incorporated, Pleasanton, CA). Nineteen scalp electrodes were used in the standard 10-10 system. Placement of iEEG electrodes was performed by two neurosurgeons (FP, SG) using the robotic stereotactic assistance device ROSA software (Rosa; Medtech Montpellier, France).

Intracranial seizure onset and offset time were determined by the reading epileptologist and confirmed by independent review (LM, MF), who adjusted onset and offset times in rare cases. Scalp EEG onset times were reviewed separately from iEEG to avoid bias. A comparison of seizure detection on intracranial data and scalp data using this data sets was previously published (22).

One hundred and two seizures of mesial temporal lobe onset were analyzed from 19 patients who had simultaneous stereo and scalp EEG as part of their evaluation for their DRE. For all seizures, the integrity of scalp and intracranial electrodes was intact. Movement artifact was not excluded. For the 19 patients included, 7 had normal imaging and 12 had abnormal imaging. Of the 12 with abnormal imaging, only one had prior epilepsy surgery (R mesial temporal laser ablation). Nine of the patients had lesions in the mesial temporal area. Three had lesions that did not localize to the mesial temporal area (cingulate cavernoma, diffuse encephalomacia, and bilateral insular/lateral temporal polymicrogryia). The patient with the polymicrogyria had seizure onsets in the mesial temporal area and arising from the insula and lateral temporal lobe. Only the seizures of mesial temporal lobe onset were included.

All patients had 19 bilateral scalp electrode contacts for analysis, placed using the standard 10-10 system. Of these 102 seizures, 35 were not seen on the scalp EEG and were surface negative. These seizures were either focal aware or subclinical. Of the remaining 67 seizures, 7 had simultaneous scalp and iEEG onset and 60 had a delayed scalp onset. Eighty seizures were of right mesial temporal onset and 22 of the seizures were of left mesial temporal onset. Sixty-eight seizures were focal aware or subclinical, 18 seizures were focal impaired aware seizures, and 16 were focal to bilateral tonic clonic. As the SEEG arrays for each patient could differ in the density of coverage and number of electrodes, a subset of SEEG contacts common to all patients was used. These were selected by visual analysis of both the SEEG and the post-operative CT to ensure electrode integrity and proper anatomic placement. For each hemisphere 24 contacts were used with 4 contacts in each of the following areas: amygdala, lateral anterior temporal, hippocampus, lateral mid temporal, medial orbitofrontal and lateral frontal. Five patients had unilateral studies (24 SEEG contacts) and the remaining 14 patients had bilateral studies (48 SEEG contacts).

### Data Processing

Our model was written in Python and run on a Macbook Pro using Spyder with Adam as the optimizer. Adam has a high performance for machine learning with high computational efficiency and little memory requirements. All EEG data was converted to EDF files without bandpass or notch filters.

Prior to running the DNN model, EEG signal segmentations were chosen from non-overlapping EEG data with the sampling rate of 512 Hz. Each similarity graph was calculated from 1 s of EEG data, i.e., the similarity between two EEG channels during 1 s was calculated from 512 data points. For DNN models with a 1 s time series, each sample is a single similarity graph that was calculated from 1 s of EEG. For DNN models using 2 or 6 s time series, each sample is 2 or 6 similarity graphs calculated from 2 or 6 consecutive time series.

### Modeling Multichannel EEG by Similarity Graphs and DNN

The relevant parameters and the notation is summarized below:

**Table:** 

***A*_*cc*_**	**Correlation Coefficient similarity graph adjacency matrix**
***A*_*MI*_**	**Mutual Information similarity graph adjacency matrix**
***A*_*waxman*_**	**Waxman similarity graph adjacency matrix**
***y*_*it*_**	***t*^*th*^ sample of the time series measured at channel *i***
**μ_*it*_**	**The mean values of *y*_*it*_**
**σ_*it*_**	**The standard deviation of *y*_*it*_**
***P*_(_*y*__*it*_, *y*_*jt*_)_**	**The joint probability mass function of *y*_*it*_ and *y*_*jt*_**
***p*_*y*_*it*__**	**The marginal probability mass function of *y*_*it*_**
***n*_*ij*_**	***L*_2_ norm of *y*_*it*_ and *y*_*jt*_**.

Samples of non-seizure, pre-seizure and seizure data were randomly extracted from our EEG data sets. The ratio of these three classes were non seizure: pre-seizure: seizure – 4:3:2. The length of time was determined by the length of the seizure. For example, if a seizure was 60 s, then 120 s of non-seizure data and 90 s of pre-seizure data was used to train the model. Based on our prior work, the pre-seizure period was defined as the 10 min prior to seizure onset.

Multi-electrode time series data were quantized into 1 s windows. For each window, a graph where nodes represent the contacts, and pairwise edges indicate a measure of similarity between the contacts was constructed. In addition to using a single second of EEG data, the interaction between consecutive time series was analyzed for 2 and 6 s time series. In order to compare the effect of different similarity metrics, we proposed 3 graph construction models based on computing the pairwise similarity between two electrodes: Correlation Coefficient, Mutual Information and Waxman model. Each graph model was tested against different DNNs to determine the combination that yielded the highest accuracy for correctly classifying EEG data as either not-seizure, pre-seizure or seizure.

#### Similarity Metrics and Graph Models

**Figure 1:**
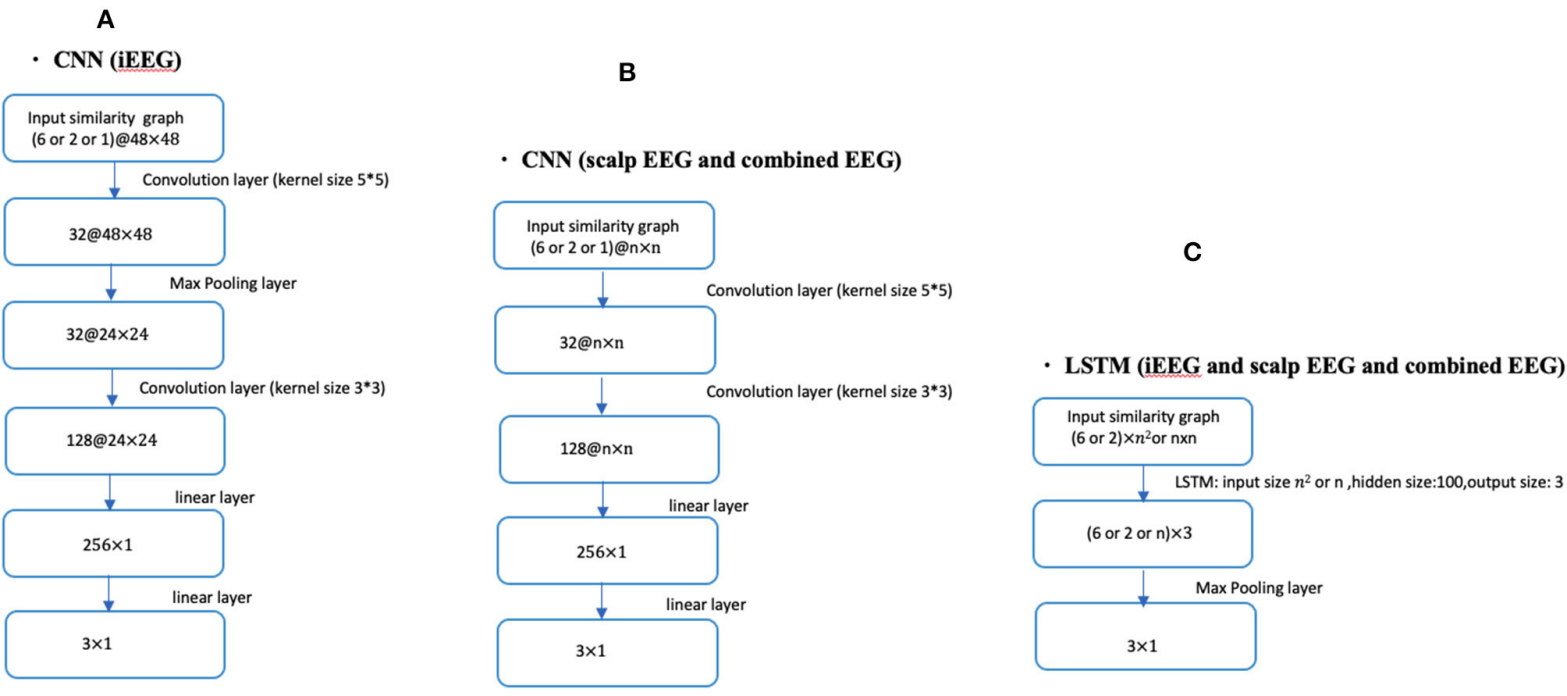
CNN and LSTM architecture using different lengths of time series (1, 2, or 6 s) in conjunction with iEEG, scalp EEG or combined EEG similarity graphs. **(A)** Shows the CNN architecture for iEEG data only. **(B)** Depicts the CNN architecture for both scalp EEG data and the combined input of scalp and iEEG data. **(C)** Shows the LSTM architecture used for all three cases of the input (i.e., iEEG, scalp EEG, and combined EEG recordings). The dimension of input similarity graph adjacency matrix was *n x n*, where *n* = 48 for iEEG data; *n* = 19 for scalp EEG data and *n* = 67 for combined EEG studies. Inputs to DNNs are vectorization of these matrices.

Fourteen patients had bilateral SEEG with 48 contacts selected while the remaining 5 had unilateral SEEG with 24 SEEG contacts. For the unilateral iEEG, the architecture was maintained with 48 contacts, 24 with recorded EEG data and 24 without data. The similarity value between the 24 non-recorded contacts was defined as 0. Similarly, the value between the recorded and the unrecorded contacts was set at 0. The dimensions of the input similarity graph adjacency matrix was *n x n*, where *n* = 48 for iEEG data; *n* = 19 for scalp EEG data and *n* = 67 for combined EEG data (Figure 1). The adjacency matrices were then vectorized as the inputs to the DNNs yielding to 2,304 (48 × 48) for iEEG, 361 (19 × 19) for scalp EEG, 4,489 (67 × 67) for joint scalp and iEEG.

Next we formally describe how to compute the similarity metrics. Consider N channels of recorded EEG signals, each channel associated with an observable time series${\left\{y_{it}\right\}}_{t=1}^{T}$ measured over T time-slots, *y*_*it*_ denotes the *t*^*th*^ sample of the time series measured at channel *i*, i.e., the EEG recording of channel *i* at *t*^*th*^ second.

1. *Correlation Coefficient* based similarity graph ${\left\{A_{CC}\right\}}_{t=\;1}^{T}$,

the similarity coefficient between channel *i* and channel *j* can given by ${\left\{c_{ij}\right\}}_{t=1}^{T}$, *i* = 1, 2, ..., *N*, *j* = 1, 2, ..., *N*


(1) $$\begin{array}{r} c_{ijt}=\frac{E\left[\left(y_{it}-\mu_{it}\right)\left(y_{jt}-\mu_{jt}\right)\right]}{\sigma_{it}\sigma_{jt}} \end{array}$$

where μ_*it*_ and μ_*jt*_ are the mean values of *y*_*it*_ and *y*_*jt*_, respectively, σ_*it*_and σ_*jt*_ are the standard deviation of *y*_*it*_ and *y*_*jt*_.

1. *Mutual Information* based similarity graph ${\left\{A_{MI}\right\}}_{t=\;1}^{T}$,

the similarity coefficient between channel *j* and channel *j* can given by


$$\begin{array}{l} {\left\{m_{ij}\right\}}_{t=1}^{T},i=1,\;2,...,N,j=1,\;2,...,N \end{array}$$

where *P*_(_*y*__*it*_, *y*_*jt*_)_ is the joint probability mass function of *y*_*it*_ and *y*_*jt*_, respectively, *P*_*y*_*it*__ and *P*_*y*_*jt*__ are the marginal probability mass functions of *y*_*it*_ and *y*_*jt*_.


(2) $$m_{ijt}=I\left(y_{it};y_{jt}\right)=\sum_{i\;\in y_{it}\;}^{\;}\sum_{j\;\in y_{jt}}^{\;}p_{\left(y_{it\;},y_{jt\;}\right)}\left(i,j\right)log\left(\frac{p_{\left(y_{it\;},y_{jt}\right)}\left(i,j\right)}{p_{y_{it}}\left(i\right)p_{y_{jt}}\left(j\right)}\right)$$

1. In *Waxman model* based similarity graph ${\left\{A_{waxman}\right\}}_{t=\;1}^{T}$,

the similarity coefficient between channel *i* and channel *j* can be given by ${\left\{w_{ij}\right\}}_{t=1}^{T}$,*i* = 1, 2, ..., *N*,*j* = 1, 2, ..., *N*.


(3) $$\begin{array}{r} w_{ijt}=\beta *exp\left(-n_{ij\;}/\alpha \cdot max\left(n\right)\right) \end{array}$$

where β = 0.4, α = 0.1, and *n*∈*R*^*N*×*N*^ have *n*_*ij*_ = || *y*_*it*_− *y*_*jt*_||_2_, *n*_*ij*_is the *L*_2_ norm of *y*_*it*_ and *y*_*jt*_.

#### Deep Neural Network Architectures for the Joint Problem of Seizure Detection and Prediction

The seizure detection and prediction problems were addressed jointly by training several different types of neural network architectures. One shallow and several deep neural network (DNN) architectures were used. A single layer neural network was constructed as a baseline comparison. For the DNNs, we focused on Convolutional Neural Networks (CNNs) and Long Short-Term Memory (LSTM) and reported the classification accuracy for the 3 class classification problems. Figure 1 shows the overall DNN architecture used in this study with different layers and input dimensions depending on the data sets being analyzed (intracranial, scalp, or combined). We trained the CNNs by using the Adam optimizer with 0.0005 as the learning rate, and we trained the LSTM by using Adam optimizer with 0.001 as the learning rate.

#### The Four-Class Classifier System for Lateralizing Seizure Onset Using Scalp EEG

In order to analyze anatomic localization, the 3-class classifier system (non-seizure, pre-seizure, and seizure) was expanded to a 4-class classifier system with the seizure category sub-divided into left and right mesial temporal onsets. Compared with the DNN architectures shown in Figure 1, we extended the dimension of the output layer to 4 × 1. For the 4-class classifier system, we trained the CNN (1 s time series) in conjunction with the Waxman graph by using the Adam optimizer with 0.0005 as the learning rate, then presented the classification accuracy using scalp EEG data. Seizures from both the bilateral SEEG and unilateral SEEG studies were included. For the unilateral SEEG, the ground truth onset lateralization was assumed to be the side with the electrodes implanted. In no cases did the scalp data or semiology suggest a different lateralization.

## Results

### Seizure Detection and Prediction

The data was split into a training set and a testing set in multiple analyses. The training and test sets for each learning model were kept separate to prevent data snooping. Furthermore, we performed 5-fold cross validation to ensure there is no overfitting. Seizure onset time was defined as the time of the onset on iEEG data as the scalp onset was often delayed and in 35 seizures not present at all (surface negative seizures).

In the first analysis, all epochs from 16 patients were used as the training set and all epochs from the remaining 3 patients as the testing set. This process was repeated 5 times (5-fold cross validation) to ensure that the 3 patients in the testing set were different. Table 1 reports the average accuracy on the testing sets for iEEG data alone, Table 2 shows the results with the same approach on scalp EEG data alone, and Table 3 reports the results on the joint iEEG and scalp EEG data sets. Within the training and test data sets epochs are treated as a *bag-of-epochs*.

**Table 1:** Impact of different combinations of similarity graphs with a shallow NN or DNNs on the average accuracy of intracranial EEG classification with 5-fold cross validation using 16 patients in the training set, and 3 patients in the testing set with multiple random splits.

**Intracranial EEG**	**Base line: Shallow NN (1 s time series)**	**CNN (6 s time series)**	**CNN (2 s time series)**	**CNN (1 s time series)**	**LSTM (6 s time series)**	**LSTM (2 s time series)**	**LSTM (1 s time series)**
Correlation Coefficient graph	90.14%	97.10%	97.96%	99.14%	90.16%	94.40%	99.27%
Waxman graph	90.48%	97.93%	98.20%	99.38%	93.48%	96.48%	98.61%
Mutual Information graph	88.12%	95.08%	96.19%	97.13%	90.10%	93.30%	96.39%

**Table 2:** Impact of different combinations of similarity graphs with a shallow NN or DNNs on the overall average accuracy of scalp EEG classification with 5-fold cross validation using 16 patients in the training set, and 3 patients in the testing set with multiple random splits.

**Scalp EEG**	**Base line: Shallow NN (1 s time series)**	**CNN (6 s time series)**	**CNN (2 s time series)**	**CNN (1 s time series)**	**LSTM (6 s time series)**	**LSTM (2 s time series)**	**LSTM (1 s time series)**
Correlation Coefficient graph	87.99%	95.66%	96.95%	97.76%	89.32%	93.65%	97.93%
Waxman graph	88.36%	97.08%	97.50%	98.56%	92.54%	95.28%	97.87%
Mutual Information graph	85.42%	90.01%	90.36%	92.14%	89.43%	90.22%	91.23%

**Table 3:** Impact of different combinations of similarity graphs with a shallow NN or DNNs on the overall average accuracy of iEEG and scalp EEG classification with 5-fold cross validation using 16 patients in the training set, and 3 patients in the testing set with multiple random splits.

**Intracranial and Scalp EEG**	**Base line: Shallow NN (1 s time series)**	**CNN (6 s time series)**	**CNN (2 s time series)**	**CNN (1 s time series)**	**LSTM (6 s time series)**	**LSTM (2 s time series)**	**LSTM (1 s time series)**
Correlation Coefficient graph	88.43%	96.69%	98.16%	98.25%	89.96%	94.39%	98.42%
Waxman graph	88.66%	97.45%	98.11%	98.99%	93.15%	96.14%	98.65%
Mutual Information graph	87.95%	94.82%	96.30%	97.21%	90.70%	93.66%	96.10%

Classification accuracy was repeated on iEEG data alone using a patient agnostic approach (Table 4). For this analysis, all epochs were separated into 5 groups with 4 of those groups used for training and the remaining epochs used for testing. This was performed a total of 5 times (5-fold cross validation) with epochs randomly assigned to one of the 5 groups. Table 4 reports the average accuracy on the testing set. This approach mixed the data from all the patients and treated the data as a *bag-of-epochs*.

**Table 4:** Impact of different combinations of similarity graphs with DNNs on the overall average accuracy of intracranial EEG classification with 5-fold cross validation based on patient agnostic epochs.

**Intracranial EEG**	**CNN (6 s time series)**	**CNN (2 s time series)**	**CNN (1 s time series)**	**LSTM (6 s time series)**	**LSTM (2 s time series)**	**LSTM (1 s time series)**
Correlation Coefficient graph	97.07%	98.01%	99.13%	90.10%	94.38%	99.32%
Waxman graph	97.97%	98.21%	99.38%	93.46%	96.51%	98.60%
Mutual Information graph	95.13%	96.27%	97.14%	90.06%	93.32%	96.44%

The classification accuracy was nearly identical with this patient agnostic approach. The rest of the discussion focuses on the results presented with each patient's data being kept as a whole in either the testing or training set (Tables 1–3).

For all EEG data, results were poorest (as expected) when the single layer (shallow) neural network was used. When using the DNNs, for the iEEG data, the least accurate model was the LSTM (6 s time series) with the Mutual Information graph at 90.10% and the highest accuracy was 99.38% using CNN (1 s time series) in conjunction with the Waxman graphs. This latter combination was the most accurate for scalp (98.56%) as well as for the combined data sets (98.99%). While accuracy was high for all EEG subsets, the accuracy was highest for iEEG (99.38%) and lowest for scalp EEG (98.56%). All subsequent analysis is based on CNN (1 s time series) with the Waxman graphs.

**Figure 2:**
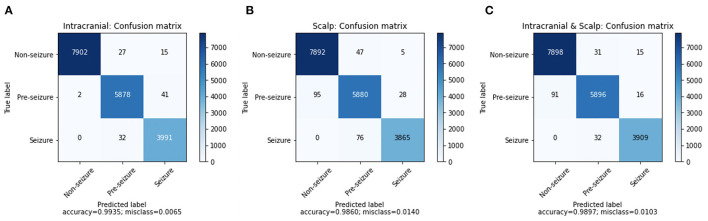
The confusion matrix of CNN (1 s time series) with Waxman graph of **(A)** iEEG data, **(B)** scalp EEG and **(C)** scalp and iEEG data jointly.

A confusion matrix for iEEG, scalp EEG and all EEG (Figure 2) demonstrates how many time windows in each class can be correctly classified as well as the specific errors of misclassification. The confusion matrices are obtained by using CNN model with the Waxman graphs constructed in 1 s windows. For example, in Figures 2A, **4**, 023 s of IEEG seizure were inputted into the model and classified correctly as seizure for 3,991 s and misclassified as pre-seizure for 32 s.

The misclassification percent was 0.65% for iEEG, 1.40% for scalp, and for combined data it was 1.03%. The model showed a low false positive rate with high classification accuracy for non-seizure EEG (99.47% iEEG, 99.34% scalp, 99.42% all EEG). The pre-seizure EEG epochs were correctly classified 99.27% (iEEG), 97.95% (scalp EEG) and 98.2% (all EEG) of the time. For seizure, the data was classified correctly in 99.20% (iEEG), 98.07% (scalp EEG), 99.19% (all EEG). Interestingly, EEG epochs from seizures were rarely misclassified as pre-seizure but were **never** misclassified as non-seizure.

### Seizure Detection From Scalp EEG

**Figure 3:**
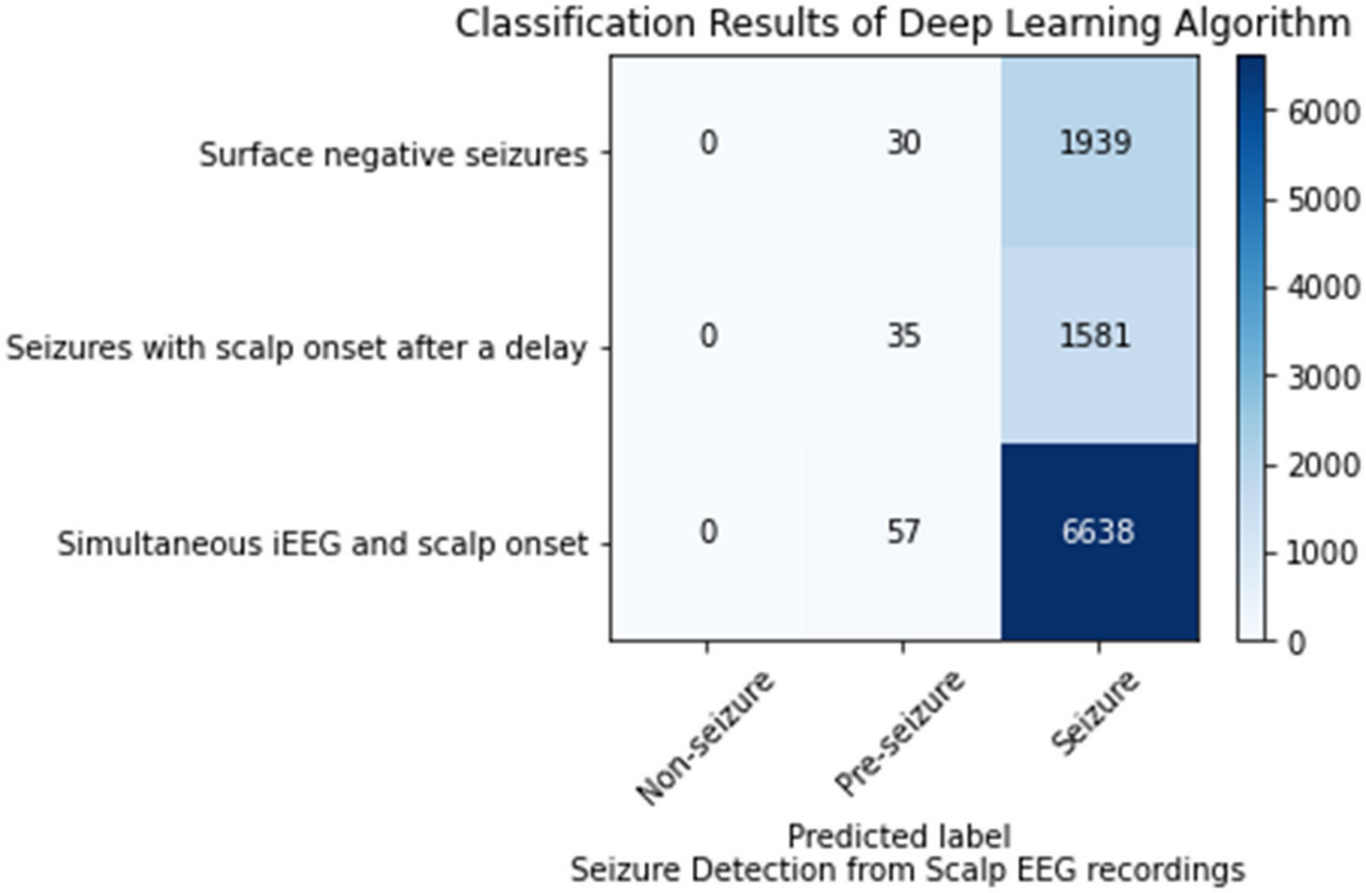
Seizure detection on scalp EEG data for seizures that are surface negative, have an onset after the iEEG onset (only surface negative portion of seizure inputted), and have a simultaneous scalp and iEEG onset.

This analysis sought to ascertain if the model could detect the seizures or the portions of seizures that were not visible on scalp EEG. Of the 102 seizures analyzed 35 were surface negative (not seen on scalp EEG), 60 were seen on scalp EEG after a delay, and 7 had simultaneous iEEG and scalp onsets. For the surface negative seizures, the CNN model (1 s time series) in conjunction with the Waxman graph detected the seizure 98.47% of the time. For the 60 seizures with a delayed scalp onset, only the scalp EEG prior to a visible seizure was used in this analysis, essentially the surface negative portion of the seizure. The model classified these seizures correctly 97.83% of the time. The seizures with a simultaneous iEEG and scalp EEG onset were classified correctly classified 99.1% of the time. Any misclassifications labeled seizure data as pre-seizure, none as non-seizure, as shown in Figure 3.

### Lateralization of Seizure Onset From Scalp EEG

**Figure 4:**
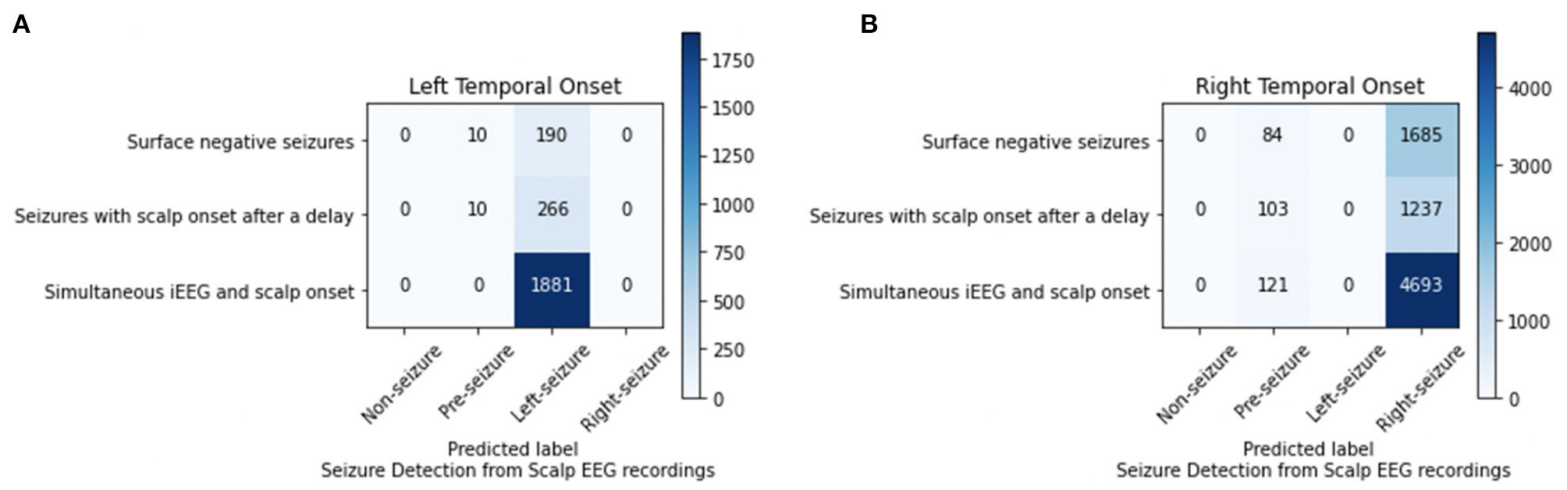
Analysis of left **(A)** and right **(B)** mesial temporal onset seizures using scalp EEG data for seizures that are surface negative, have an onset after the iEEG onset (only surface negative portion of seizure inputted), and have a simultaneous scalp and iEEG onset.

For the 4-class classifier system (non-seizure, pre-seizure, left-seizure, right-seizure), the accuracy of the CNN (1 s time series) in conjunction with the Waxman graph was 98.11% with scalp EEG as the input. Seizure detection accuracy was high. For the seizures of left mesial temporal onset, surface negative seizures were classified correctly 95.00%, the surface negative portion of seizures with a delayed onset 96.38% and the simultaneous onset seizures 100%. For the seizures of right mesial temporal onset, surface negative seizures were classified correctly 95.25%, the surface negative portion of seizures with a delayed onset 92.31% and the simultaneous onset seizures 97.49% (Figure 4). Importantly, seizures of left brain onset were never misclassified as right brain onset and the reverse is true as well. This finding held firm when analyzing surface negative seizure and the surface negative portion of seizures with delayed scalp onset. All misclassifcation errors occurred with seizure data being mislabeled as pre-seizure data. Seizure data was never misclassified as non-seizure. For surface negative seizures (including the surface negative portion of the seizures with a delayed scalp onset), the model was able to both detect and to lateralize them with very high accuracy, as shown in Figure 4.

## Discussion

In this paper we re-explore seizure prediction and detection on a unique data sets of simultaneous iEEG and scalp EEG. As part of this work, various combinations of DNNs (both CNNs and LTSM) were tested with different similarity graph models (Correlation Coefficient, Mutual Information, and Waxman) to determine which combination had the highest classification accuracy. Interestingly, the models which used consecutive time series, 2 or 6 s, did not perform as well as the models that used a single second of EEG data for input. This is perhaps because for the longer time series there was some averaging of features which may have impacted accuracy. All models performed quite well, but the CNN (1 s time series) in conjunction with the Waxman similarity graph performed the best. This combination became the primary machine learning model in this paper.

The accuracy for correctly classifying EEG data into non-seizure, pre-seizure and seizure was over 98% for iEEG alone, scalp alone and iEEG and scalp combined. This classification system can be used for both prediction and detection. In the field of seizure forecasting and prediction, false positives have the potential to create unnecessary anxiety and intervention. In this model, the false positive rate was very low, with <1% of the non-seizure epochs being classified as seizure or pre-seizure. Further, the results suggest that prediction is indeed possible, as over 97% of EEG epochs from the 10 minutes prior to a seizure were labeled as pre-seizure. This worked best on iEEG data alone (99.27%) and slightly less well on scalp EEG data (97.95%). Correct classification of EEG data into seizure was similarly highly accurate (>98%), demonstrating high efficacy in seizure detection.

It is important to remember that this 10 min pre-seizure period is not different from the non-seizure period to the human eye either intracranially or on scalp. Interestingly, seizure prediction accuracy was not very different than seizure detection accuracy; even though for the experienced epileptologist detecting a seizure is quite easy and predicting a seizure is impossible.

Surface negative seizures are seizures that do not appear electrographically on a scalp EEG but are visible intracranially. These can be clinical or subclinical. If clinical, they are usually focal aware seizures, like focal motor or a temporal lobe aura. In our previous work with simultaneous scalp and iEEG electrodes, 67% of focal aware and 67% of subclinical seizures had no visible scalp EEG seizure (22). This can occur when the seizure involves <6 cm^2^ of cortex and/or when the source is deep. In this work, 35 of the seizures were surface negative and 60 of the seizures had a surface negative portion (intracranial onset occurred first followed by a delayed scalp EEG onset). *The model was able to classify these surface negative seizures accurately as seizures over 97% of the time using scalp EEG data alone*. This suggests that seizure detection is possible using scalp EEG alone, even when the seizure is not visible to the human eye.

Lastly, the model was able to *successfully lateralize scalp EEG data into left and right onset* (all were mesial temporal) with high accuracy, using a 4-class DNN classifier. This is perhaps not surprising for seizures that are visible on scalp. More interesting is that this accuracy held firm for the surface negative seizures and the surface negative portion of the seizures with a delayed scalp onset.

In summary, the contributions of this paper are two-fold. From the neuroscience perspective, we are the first to use machine learning to (i) model, analyze, and evaluate iEEG and scalp EEG jointly, (ii) detect surface negative seizures on the scalp using scalp data, (iii) lateralize seizures using machine learning from scalp EEG data, even those that are not visible on the scalp EEG. This work expands on our previous results reported in (22).

From the machine learning perspective, our contribution is on the spatial, frequency and temporal modeling of EEG data using graph theory. In our previous work we introduced the concept of graph theoretical analysis of scalp EEG recordings for seizure prediction and detection using hand crafted features (19, 21). In this work we (i) introduce and analyze different similarity metrics for graph construction, and (ii) use the graph adjacency matrices as the input to deep learning algorithms (CNN and LSTM) which extract and learn from convoluted graph features. To our knowledge, we are the first to apply the Waxman model to the seizure prediction problem. This model is a method of determining if two nodes on a graph are linked (20) and has been used in communication and data networks. It surprisingly outperformed the other two similarity graphs tested, suggesting future utility in EEG modeling.

### Clinical Significance

To our knowledge, this is the first work to provide seizure prediction and detection using machine learning on a combined data sets using simultaneous iEEG and scalp EEG. The iEEG seizure onset time was used as the ground truth. Accuracy for both prediction and detection was high whether or not the input was iEEG data alone or scalp EEG data. This suggests that different devices could be constructed from different sources of EEG data depending on the clinical need. A wearable extracranial seizure prediction device may be of use for a person with rare but dangerous seizures who wishes to do a higher risk activity like hiking. While a permanent intracranial prediction device would be of greater use for people with refractory epilepsy and more frequent seizures.

The ability to detect surface negative seizures from scalp data may provide additional opportunities to non-invasively understand surface negative seizure frequency and impact. Both predicting a seizure before it occurs and detecting seizures at their onset, before they manifest on scalp EEG, suggest a window for intervention. Possible interventions include administration of a fast acting medication or simply getting into a safe position and notifying family.

The model was able to successfully lateralize all seizures, even those that were not visible on scalp EEG. This suggests that it may in the future be possible to detect surface negative seizures in the epilepsy monitoring unit and lateralize them, which has the potential to shorten length of stay. Additionally, accurate lateralization can help guide surgical work-up and management and may give greater detail to the seizure network, the visible and the invisible.

### Future Research and Limitations

The study was limited by data that was retrospective and from only 19 patients. Additionally, we purposefully limited this paper to seizures of mesial temporal onset for a more homogenous group. However, it is not known if these results can be generalized to seizure onsets in other parts of the brain. While this work shows accurate lateralization, a more intensive study of localization using seizures of different onset location would be of value.

In this paper, each 1 s window was treated as independent, but for a real-time deployment of a prediction or detection system, a risk assessment model, which considers the labeling of consecutive 1 s windows, can be developed using conditional probabilities. In other words, if the model assigns a 1 s epoch into a category, the risk assessment model may require several consecutive seconds to be classified similarly before the system makes a categorization determination. This will decrease the false positive rate. The next planned project is to test this program prospectively on patients with simultaneous intracranial and scalp EEG undergoing an epilepsy surgical work-up.

Accurate seizure prediction and detection will enable the creation of wearable and implantable devices. In recent work on seizure prediction using scalp EEG, there have been advancements that will make it easier to deploy within hardware (23–25). A limitation of the paper is that we did not use other algorithms on our data sets for direct comparison. We met our goal with high accuracy of classifying EEG data including demonstrating it is possible to detect seizures on scalp EEG that are not visible. Future research will need to allow for direct comparisons as well as refinement of methods in order to optimize models for use in portable devices.

The work in seizure prediction does indicate a pre-seizure state, during which a seizure is nearly inevitable. However, the transition from non-seizure to pre-seizure is not understood. One avenue of research is to investigate the DNN themselves by creating topographical images of the model, ie saliency maps, to further inform us as to the nature of the pre-seizure state. A very different avenue is to use prediction tools to conduct real time experiments during that pre-seizure period of minutes to understand the biology of the transition into seizure, and the epileptic brain.

## Data Availability Statement

The data sets presented in this study can be found in online repositories. The name of the repository and accession number(s) can be found below: Rensselaer Polytechnic Institute (RPI) Data Science Research Center (DSRC), http://dsrc.rpi.edu/?page=databank. Requests to access these data sets should be directed to Daniel Park, parkd5@rpi.edu.

## Ethics Statement

The studies involving human participants were reviewed and approved by Icahn School of Medicine Institutional Review Board. Written informed consent for participation was not required for this study in accordance with the national legislation and the institutional requirements.

## Author Contributions

ZL, MF, LM, and BY conceived and designed the project. ZL and BY carried out all computational analysis. MF and LM provided the data, domain expertise, ground truth, and interpreted the computational analysis. ZL, BY, MF, LM, FP, and SG contributed to the writing of the manuscript. All authors contributed to the article and approved the submitted version.

## Conflict of Interest

The authors declare that the research was conducted in the absence of any commercial or financial relationships that could be construed as a potential conflict of interest.

## Publisher's Note

All claims expressed in this article are solely those of the authors and do not necessarily represent those of their affiliated organizations, or those of the publisher, the editors and the reviewers. Any product that may be evaluated in this article, or claim that may be made by its manufacturer, is not guaranteed or endorsed by the publisher.
